# Advising Parents in the Face of Scientific Uncertainty: An Environmental Health Dilemma

**DOI:** 10.1289/ehp.119-a436

**Published:** 2011-10-01

**Authors:** Naomi Lubick

**Affiliations:** Naomi Lubick is a freelance science writer based in Stockholm, Sweden, and Folsom, CA. She has written for *Environmental Science & Technology*, *Nature*, and *Earth*.


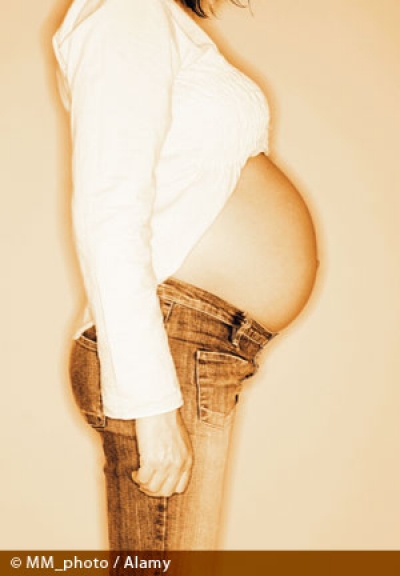
Naomi Stotland and her colleagues have been trained to tell their obstetric patients not to blame themselves for a miscarriage; fetal genetic abnormalities and other uncontrollable factors are generally the cause.^1^ But after listening to one of her residents counsel a patient who had just had a miscarriage, “I asked myself if we were leaving out part of the story,” Stotland wrote in a recent blog post.^2^

For Stotland, a researcher with the Program on Reproductive Health and the Environment (PRHE) at the University of California, San Francisco (UCSF), the story includes where patients live and work—for instance, if they have jobs where they must use solvents, say, as a housecleaner or working at a dry cleaners or in a nail salon. Environmental health research links occupational solvent exposures to adverse health outcomes including miscarriage and birth defects,^3^^,^^4^ but doctors may not ask their patients whether they encounter these chemicals, instead focusing on warning them about alcohol and drug use or asking if they smoke, Stotland wrote.^2^

Although population-level effects have been documented for many pollutants, direct connections between environmental exposures to chemicals and individual health outcomes can be hard to make, for both clinicians and laypeople. These seemingly spectral threats may be the least of some parents’ worries, and for that matter, their doctors’ as well. For other parents, these issues are a source of great concern, even though the potential health effects involved for individuals are far from certain.

Jeanne Conry, chairwoman of the American Congress of Obstetricians and Gynecologists for California, says some doctors face enough challenges in managing well-known problems such as the rise in diabetes and obesity in expectant mothers.^5^ “The risk of birth defects if [a pregnant woman’s] blood sugar gets out of control is significant, several times higher than somebody who has normal blood sugars,” she says. “The research has been out there for years, and we’ve been trying to get women to understand that kind of thing . . . [but] we haven’t accomplished that.” With such established worries, she surmises, less well-defined environmental threats may be considered secondary risks by most practicing doctors.

**Figure:**
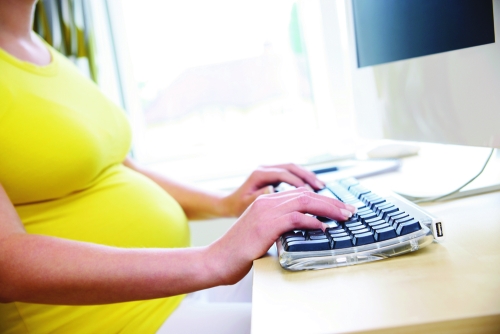
It takes time to get the message out. You have to do this in a multipronged approach. *—Jeanne Conry, American Congress of Obstetricians and Gynecologists for California* © 2011 Thinkstock

But the many uncertainties around specific environmental health hazards should not be confused with the strength of the evidence, says Patrice Sutton, a research scientist at PRHE. She and her PRHE colleagues therefore seek to provide evidence-based information in a responsible manner to doctors and other clinical practitioners who serve as a frontline for pregnant women and other patients concerned about exposure to environmental chemicals. Their ultimate target is the larger community and the legislators and policymakers who could regulate chemical exposures.

“Our approach is to communicate the science clearly so that individuals have the information they need to apply their values and preferences,” Sutton says. But communicating about environmental exposures implies more than changing individual behaviors, she adds: “Because many exposures are not avoidable by individuals, it means harnessing knowledge to improved policy to prevent the introduction of hazardous exposures into homes, communities, and workplaces.”

## Handling Complexity

For those accustomed to the caveats of environmental health research, the concepts presented by the PRHE team and groups like them are not surprising: People’s bodies carry measurable amounts of environmental chemicals. Many of these chemicals are transferred directly across the placenta to the developing fetus and through breast milk to the nursing child. But for ethical reasons it is usually not possible to directly test for health effects in humans, so toxicologic research often depends on *in vitro* and animal studies. That can introduce scientific uncertainty about the direct health effects for humans.

Meanwhile, absent regulatory policy or clinical guidance, the decision to avoid these chemicals or not remains up to individuals, who often must educate themselves and make choices alone. Hannah Gardener, a consultant with a PhD in epidemiology from Harvard University, says her background in science gives her “the benefit of being knowledgeable”—she can check PubMed, read the peer-reviewed literature, and come to her own conclusions from a scientist’s perspective. Average consumers can do the same, in theory, but they may be intimidated by or unable to interpret the scientific terminology, and other information online may not be as accurate, she says.

“You cannot expect families to know [about the latest in toxicity testing],” says Bruce Lanphear of Simon Fraser University and a part-time researcher at Cincinnati Children’s Hospital. “Even as somebody who studies something like this for a living, it is virtually impossible for me to keep up” with all the new research for both old chemicals and new products emerging on the scene. Consumers have more than enough to worry about on their own, like the current recession, he says.

So how can clinicians advise pregnant women and others on environmental exposures in an accurate, responsible manner? Communicating about potential hazards requires delicacy and clarity, the PRHE researchers and others say, with full acknowledgment of the limits of current scientific understanding.

Joanne Perron, a former ob/gyn now studying occupational and environmental medicine as a UCSF postdoctoral fellow, says one of her self-enforced rules is to “not turn into an Eeyore when I discuss these issues,” in order to avoid overwhelming her audience. She also says that personalizing the message and talking about her own experiences—working in a hospital, parenting her teenage sons, undergoing breast cancer treatment—also helps people comprehend and care more about environmental exposures as a personally relevant issue.

For many pregnant women, keeping track of everything involved in giving their children the healthiest start possible—including being aware of potentially hazardous environmental exposures—can be “really overwhelming,” says Heather Stapleton, a Duke University researcher who studies polybrominated diphenyl ether (PBDE) flame retardants in blood and consumer products. As a scientist communicating about her work, Stapleton says, “I want to provide as much information as possible, but I don’t want to alarm [pregnant women and mothers].”

Breastfeeding is one good example of this balance. Stapleton is well aware of the hazards associated with PBDEs in animals^6^ (human health effects are less clear) and the ease with which they are transferred through breast milk.^7^ Yet, she says, “being a mom, I still make the choice to breastfeed my children.” She emphasizes that breastfeeding carries innumerable immune system benefits, and scaring mothers away from it on the basis of potential chemical exposures to their children could, ironically, be detrimental to their kids’ health.

Tom Webster, associate chair man of the Department of Environmental Health at the Boston University School of Public Health and one of Stapleton’s collaborators, notes that nurses and other clinicians have worried that telling women about the chemicals in their bodies, particularly in breast milk, could discourage them from breastfeeding. But he and his colleagues recently reported otherwise: women continued to breastfeed even after participating in a study to measure PBDEs in their milk and getting reports on their body burdens.^8^

“People want to know,” says Lanphear, who recently coauthored a report on the preferences of participants in a biomarkers study of more than 300 mothers.^9^ Nearly every mother wanted to see results for different contaminants in herself and her child. The team presented the individual test results along with national averages from the National Health and Nutrition Examination Survey, while providing information about how to reduce certain environmental exposures. However, very few study subjects contacted the hotline set up by the researchers to find out more about the potential health impacts of their body burdens, Lanphear says. The team also reported that the majority of participants said they preferred to contact their doctors for this kind of information.

“We were reassured that we weren’t creating anxiety by providing them with test results for environmental contaminants—if we were, we would expect more of the moms to contact us,” Lanphear says. “People can handle complexity, but what they do with it is a whole different matter.”

## Consumer Advice: Don’t Panic

One way environmental health scientists can communicate directly with the public is by providing credible information online for people seeking it there. For example, readers might stumble across the blog where Stotland posted her essay on miscarriage. Called The Clinic, and hosted on Mission Loc@l (a San Francisco–based online news website run by the journalism program at the University of California, Berkeley), the blog contains essays by PRHE researchers and program fellows on topics such as possible links between estrogenic chemicals and uterine fibroids, the decision whether to buy organic or conventional produce, and placental transfer of chemicals.

**Figure:**
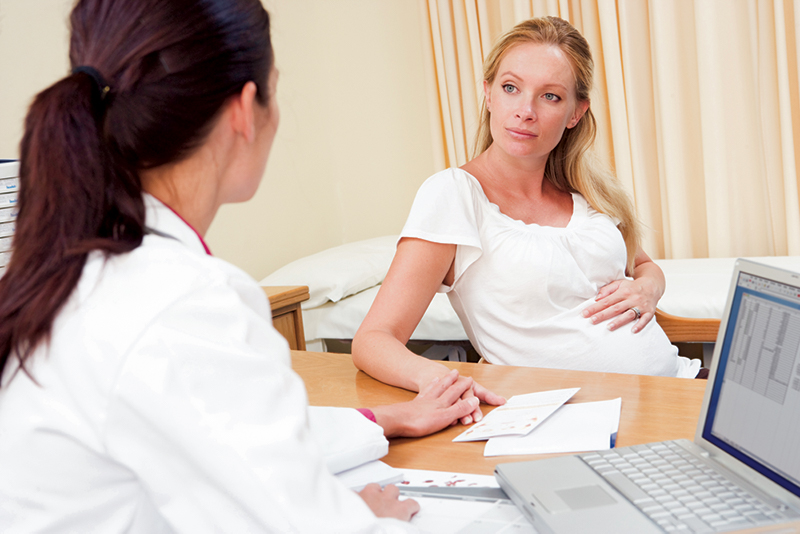
Our approach is to communicate the science clearly so that individuals have the information they need to apply their values and preferences. . . . Because many exposures are not avoidable by individuals, it means harnessing knowledge to improved policy to prevent the introduction of hazardous exposures into homes, communities, and workplaces. *—Patrice Sutton, University of California, San Francisco* © 2011 Thinkstock

Online media may work for younger audiences that have grown up with Facebook, comments Webster. But getting the messages out requires using every avenue available, which might mean newspapers or other traditional media for some readers. He says that as a scientist, he tries to broadcast his most important findings to journalists if he can.

In her capacity as a consultant, Gardener assesses homes for potentially toxic products and conditions. In her home consulting visits, she suggests easy substitutes known to be nontoxic or other solutions, such as replacing very old vinyl miniblinds (purchased before the late 1990s), which could release lead.^10^ Gardener says that, although her clients sometimes report being scared of what she might find in their homes, she counsels that we live in a world where we can’t eliminate everything. “We are exposed to things that are bad for us all the time,” Gardener says, but the human body is very adept at recognizing toxicants and healing the damage they cause. The message she focuses on is “optimizing health” by pursuing simple, precautionary paths without obsessing about unwittingly coming into contact with something toxic.

To gauge what practitioners know about the impact toxic chemicals can have on their patients’ health, PRHE is currently partnering with the American Congress of Obstetricians and Gynecologists to survey its membership across the country and to raise awareness of environmental health issues. Questionnaires went out in July 2011; the results, expected later this year, will be submitted to peer-reviewed journals for publication and used to inform PRHE’s clinical outreach and education efforts, according to Sutton.

Meanwhile, the medical specialists from whom pregnant women might expect to get this kind of information don’t usually receive training in environmental health issues, Perron says. Obstetricians, nurses, and other health professionals tend to approach prenatal care from the perspective of well-established harmful practices and exposures. For instance, a slew of books based on studies of thousands or tens of thousands of patients document the impacts of using various pharmaceuticals during pregnancy and lactation. “Those publications are our bible,” Conry says. “Someone comes in and says, ‘I’m taking cold medicines and just found out I’m pregnant.’ I can look up the outcomes right there.”

**Figure:**
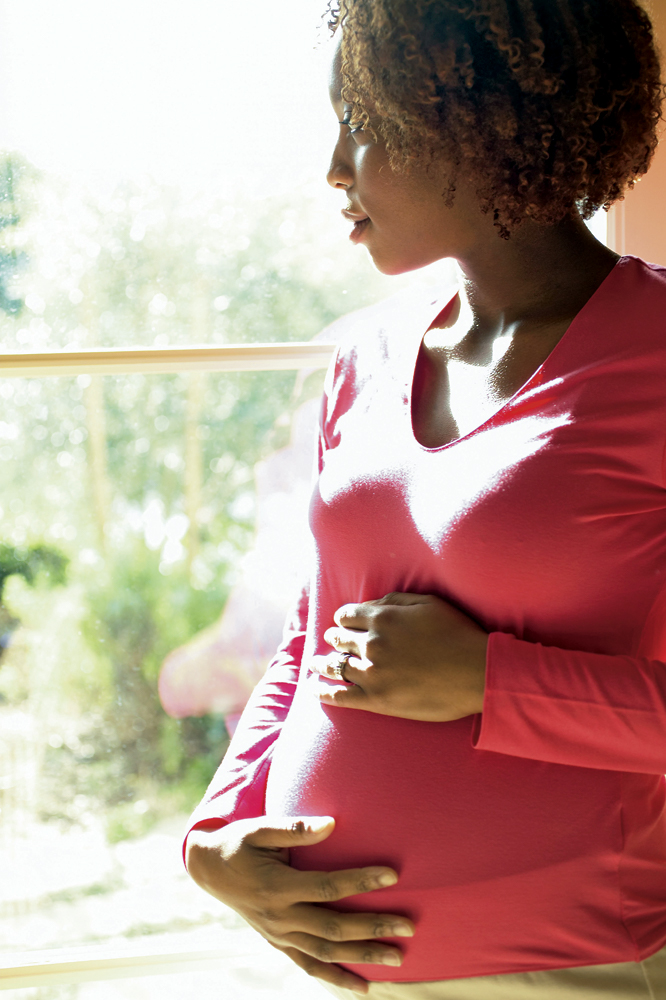
I want to provide as much information as possible, but I don’t want to alarm [pregnant women and mothers]. *—Heather Stapleton, Duke University* © 2011 Thinkstock

PRHE is partnering with California’s statewide biomonitoring program to measure more than 100 chemicals in several dozen mother–infant pairs and see if they can tease out primary routes of exposure and potential relationships between exposures and birth outcomes.^11^ Synthesizing this kind of work with other relevant research could lead to what Conry calls “a green bible,” with the same statistical rigor applied to outcomes of chemical environmental exposures as to those of pharmaceuticals. But creating any guidance that has that kind of certainty will take a long time, she comments, because the research is so difficult, particularly when striving for conclusive evidence.

But even once people know about environmental exposures, other underlying issues such as financial insecurity can thwart efforts to change behaviors and consumer choices, PRHE researchers and others point out. The price of organic versus conventional food, for instance, raises questions about the affordability of precautionary avoidance. Moreover, women who may be the most highly exposed to environmental chemicals—as workers in nail salons or dry cleaners, in agricultural fields, or in health care settings—may not be in a financial position to leave those jobs or may fear employer retaliation if they try to access or exercise their legal rights to a safe workplace, PRHE researchers say.

“We are not currently conducting research directly related to male exposures, but that is not because it is not an important area,” Sutton adds. “Although our research is on pregnancy and chemicals broadly, preconception is the time to intervene and prevent both male and female exposures.”

Clinicians can help prevent harmful exposures by asking patients about their work settings and by encouraging them to quit smoking and to remove toxic chemicals from their homes. But the workplace is an especially challenging environment because toxic exposures can be much higher in an occupational setting than in community settings. In fact, most workplace chemicals that can harm pregnancy or the developing fetus do not have protective exposure limits, Sutton says. However, knowledgeable clinicians can play an essential role in helping their patients find the medical and legal expertise they need to learn about their options and make an informed decision in the face of uncertainty.

## Expanded View

Conry sees PRHE’s efforts to communicate the problems as a first step toward long-term, high-level measures to reduce toxic exposures among the general public. She also cites positions taken by influential organizations on various environmental health issues, which largely encourage the use of the precautionary principle for avoiding unnecessary exposures to chemicals that could create human reproductive problems, even if incontrovertible proof is still forthcoming.^12^^,^^13^^,^^14^^,^^15^ Despite these powerful statements, “it takes time to get the message out,” she says. “You have to do this in a multipronged approach.”

**Figure:**
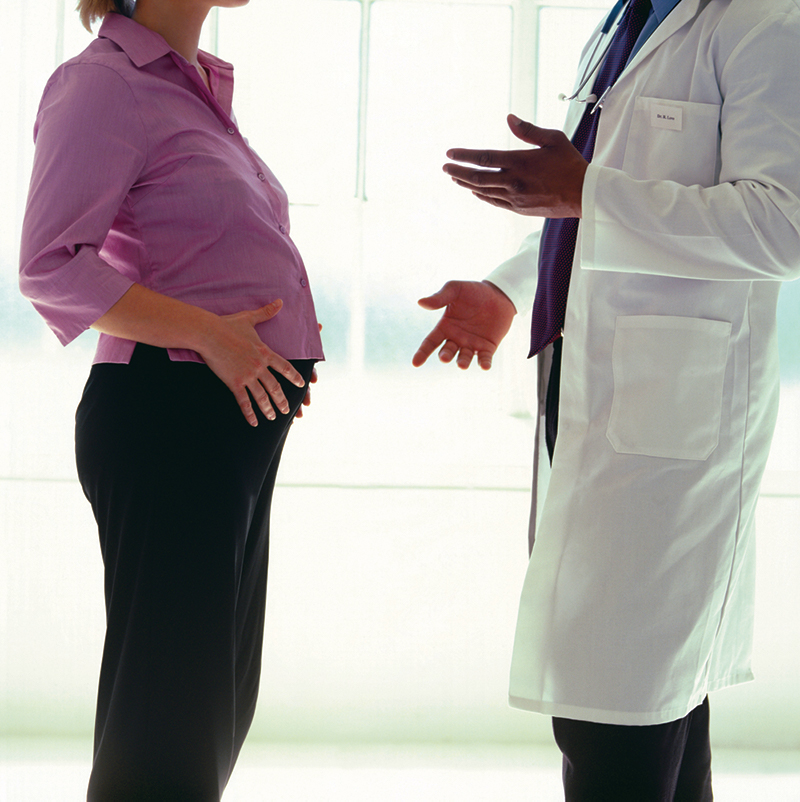
People can handle complexity, but what they do with it is a whole different matter. *—Bruce Lanphear, Simon Fraser University* © 2011 Thinkstock

Tracey Woodruff, PRHE’s director and a researcher at UCSF, wants the UCSF program to take these messages not only to patients, who can act immediately for their own comfort level, and to clinicians, who can advise at the individual patient level, but community-wide. PRHE “work[s] to train scientists, community members, clinicians, how to interpret science,” Woodruff says, and “how to reach the decision makers.”^16^

The group trains fellows every year, an even mix of doctors, nurses, and other clinicians, members from community-based organizations, and researchers.^17^ “A very important part of all of our research translation and training efforts is the message ‘make the government work for you,’” says Sutton. That means training people about the science so they can knowledgeably participate in decision making at the local, state, and national levels. According to Woodruff, PRHE works hard to communicate to all its audiences that in order to succeed, steps to prevent toxic environmental exposures cannot not be limited exclusively to individual-level action but must also involve societal-level change.

Indeed, Lanphear says, lead and other toxicants have taught us a lesson that seems to have been forgotten: “The only way to reduce exposures is to reduce environmental contamination in air, water, consumer products—that’s what led to lower blood lead levels, not advising parents about how to reduce their child’s exposure.”

In the short term, Lanphear says, it is important to take precautionary measures such as advising pregnant women to eat fish that is low in mercury. “But if at the same time we are not taking steps to reduce mercury contamination so that our grandchildren can eat fish without worrying about it, then we have failed the public.”
